# New Analysis Method Application in Metallographic Images through the Construction of Mosaics Via Speeded Up Robust Features and Scale Invariant Feature Transform

**DOI:** 10.3390/ma8073864

**Published:** 2015-06-25

**Authors:** Pedro Pedrosa Rebouças Filho, Francisco Diego Lima Moreira, Francisco Geilson de Lima Xavier, Samuel Luz Gomes, José Ciro dos Santos, Francisco Nélio Costa Freitas, Rodrigo Guimarães Freitas

**Affiliations:** 1 Programa de Pós-Graduação em Energias Renováveis, Instituto Federal de Educação, Ciência e Tecnologia do Ceará, Fortaleza, Ceará 61.939-140, Brazil; E-Mails: geilsonx@gmail.com (F.G.L.X.); samuelluz.g@gmail.com (S.L.G.); ciro@ifce.edu.br (J.C.S.); fneliocf@ifce.edu.br (F.N.C.F.); rodrigofg@ifce.edu.br (R.G.F.); 2 Departamento da Indústria–Maracanaú, Instituto Federal de Educação, Ciência e Tecnologia do Ceará, Fortaleza, Ceará 61.939-140, Brazil; E-Mail: fcodiegomoreira@gmail.com

**Keywords:** metallography, mosaic, SIFT, images, SURF

## Abstract

In many applications in metallography and analysis, many regions need to be considered and not only the current region. In cases where there are analyses with multiple images, the specialist should also evaluate neighboring areas. For example, in metallurgy, welding technology is derived from conventional testing and metallographic analysis. In welding, these tests allow us to know the features of the metal, especially in the Heat-Affected Zone (HAZ); the region most likely for natural metallurgical problems to occur in welding. The expanse of the Heat-Affected Zone exceeds the size of the area observed through a microscope and typically requires multiple images to be mounted on a larger picture surface to allow for the study of the entire heat affected zone. This image stitching process is performed manually and is subject to all the inherent flaws of the human being due to results of fatigue and distraction. The analyzing of grain growth is also necessary in the examination of multiple regions, although not necessarily neighboring regions, but this analysis would be a useful tool to aid a specialist. In areas such as microscopic metallography, which study metallurgical products with the aid of a microscope, the assembly of mosaics is done manually, which consumes a lot of time and is also subject to failures due to human limitations. The mosaic technique is used in the construct of environment or scenes with corresponding characteristics between themselves. Through several small images, and with corresponding characteristics between themselves, a new model is generated in a larger size. This article proposes the use of Digital Image Processing for the automatization of the construction of these mosaics in metallographic images. The use of this proposed method is meant to significantly reduce the time required to build the mosaic and reduce the possibility of failures in assembling the final image; therefore increasing efficiency in obtaining results and expediting the decision making process. Two different methods are proposed: One using the transformed Scale Invariant Feature Transform (SIFT), and the second using features extractor Speeded Up Robust Features (SURF). Although slower, the SIFT method is more stable and has a better performance than the SURF method and can be applied to real applications. The best results were obtained using SIFT with Peak Signal-to-Noise Ratio = 61.38, Mean squared error = 0.048 and mean-structural-similarity = 0.999, and processing time of 4.91 seconds for mosaic building. The methodology proposed shows be more promissory in aiding specialists during analysis of metallographic images.

## 1. Introduction

Many studies have been carried out on the segmentation of digital images, which seek to overcome the limitations of the various methods currently used in specific applications. Thus, existing techniques are improved and new methods developed.

In this article, tools and methodologies are presented that enable the use of Digital Image Processing techniques on the metallographic analysis of metallic materials from metallurgical processes. The mosaic technique is used to construct scenes and environments through small images with corresponding characteristics. The final result is a larger image generated from smaller images that were processed. Thus, in accordance with the generated image, it will be possible to more thoroughly analyze and process to obtain exacting results.

Metallography is one of the most important analytical studies that aim to ensure the quality of materials in manufacturing processes and the performance of studies in the formation of new alloys and materials. Proper analysis and microscopic techniques applied to the study of metallurgical products make it possible to visualize the microscopic texture of the material, and its constituent, highlighting the various grains that are formed and the respective phases of the resulting structures, allowing evaluation based on size, shape and arrangement. According to [1], the microstructure fundamentally depends on the chemical composition and also thermal and mechanical treatments to which the metal was submitted, and according with [2] the phase, is a homogeneous portion of a particular system that has uniform physical and chemical characteristics. This analysis sometimes turns out to be complex, given that the materials have different morphologies resulting from various heat treatments applied and also the employed chemical composition. According to [3], microscopic metallography, also known as micrograph studies of metallic products, with the aid of the microscope aimed at determining the constituents and the texture of the metal product, the various grains that are formed are highlighted. The record of this analysis is carried out through images and photographs taken by the microscopes.

After preparation of the specimen, metallographic analysis is made by visual inspection. However, this analysis is not entirely reliable due to human limitations. It is because of the emergence of some variables such as fatigue, distractions, repeatability actions, lack of concentration and others external factors that can potentially cause a failure in the analysis. The flaws in this process can be observed when more than one specialist conducts the analysis of the same sample and the final results are not equal.

**Figure 1:**
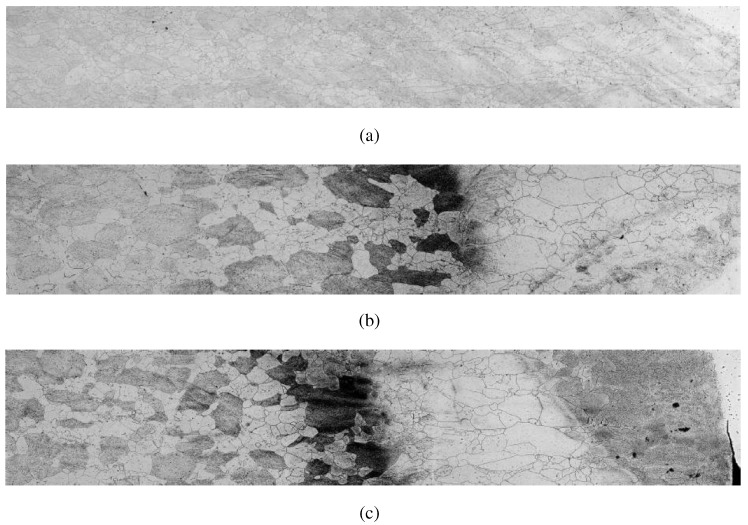
Examples of mosaics manually built for the steel microstructures metallographic analysis used in this work, (**a**), (**b**) and (**c**) are examples of samples 1, 8 and 10, respectively.

Figure 1 shows a macrograph section through weld compositions of Fe-Cr-Mo alloys with mosaic manually built. These are examples of non-commercial steel samples with 17% Cr and 5% Mo welded by MIG process with variation in energy used welding. Argon was used as shielding gas. As filler material was used ERNiCrMo-3 AWS electrode (wire with 1.2 mm diameter). Regions presented correspond to the Merged Zone (AWS ERNiCrMo 3-electrode), the heat affected zone of welding and the base material. The figures represent different points of the cross section of the joint where the welds were performed with a current of 210 A and voltage 30 V.

Microstructure following the molten zone to the base material related to the weld pass on the side of the joint.

Figure 1a was obtained with energy 9.9 kJ/cm and welding speed of 38 cm/min. Microstructure following the molten zone to the base material. Figure 1b was obtained with the energy of 19.9 kJ/cm and welding speed 19 cm/min. Figure 1c was obtained with an energy of 19.9 kJ/cm and welding speed 19 cm/min.

One of the factors that influence the emergence of failure is the fact that only small areas of the sample to be analyzed. This problem occurs because the range of microscopic vision is limited to a small area and it is impossible to analyze the entire region of the sample. This article proposes the creation of a mosaic of several adjacent metallographic images, to create one large image, allowing a global analysis to then be carried out by local analysis at specific points. The proposed model is meant to reduce the effects caused by human limitations, given that after the analysis of the overall image, only specific images can be chosen for a thorough analysis.

In metallurgy, welding technology widely uses tests and metallographic analysis. According to [3], in welding, this test allows us to know the metal features, especially in the Heat Affected Zone (HAZ); the region most likely for natural metallurgical problems to occur in welding. The expanse of the Heat Affected Zone exceeds the size of the area observed in the microscope, and typically requires several images mounted on a large picture to allow for the study of the entire Heat Affected Zone. This image stitching process is performed manually and is subject to all the flaws inherent in the human being as the result of fatigue and distraction [4,5,6].

Mosaic, in regards to Digital Image Processing, means the joining of separate and adjacent images, which are automatically grouped into a single image, the result of which is a mosaic image that is broad and consistent. Image acquisition and formation of mosaics are fairly applied in Geo-analysis because it allows the production of smaller individual images and the subsequent integration in generating a larger map. In GIS, these images are captured by remote sensors, according to [7], Image Registration is the process of aligning different images of the same scene acquired at different periods of time, different viewing angles or different sensors. This process is needed for many image processing and computer vision applications such as object recognition, robot localization, 3D medical image reconstruction and object tracking [8,9,10,11]. Traditionally, the image registration techniques used in remote sensing need to choose the Ground Control Points (GCPs) manually, at various landmarks of the images [12]. These GCPs are then used to align one image to another.

With the metallographic analysis, the mosaics will be created from images collected under the microscope using Digital Image Processing techniques and will be subsequently validated by comparing the results with the techniques currently used in conventional analysis.

The intention is to increase efficiency in the generation of results and help in decision making; considerably reducing the time of construction of the mosaics and the likelihood of errors in the group of images.

One of the challenges faced is to join several different images together in order to form a larger image with the least distortion because it will be used as an automatic generation tool for mosaics using Digital Image Processing techniques.

## 2. Materials and Methods

This section presents the database of images and its acquisition form. In addition, the Scale Invariant Feature Transform (SIFT) and Speeded Up Robust Features (SURF) methods will be presented, with their variations, used in the mosaic construction and the adoption of mosaic quality metrics.

The high level of naphthenic acidity present in oils processed in refineries requires a high corrosion resistance of equipment and ducts. Among materials used in the Petroleum industry, we highlight the Cr-Mo steels and austenitic stainless steel with molybdenum in their composition. These steels have not shown satisfactory performance when in contact with Petroleum rich in naphthenic acid. Iron-Chromium steels with Mo in their composition become an alternative to this application. The images used in this paper are from a study that analyzes the effect on the increasing the molybdenum content of the naphthenic corrosion resistance, microstructural changes, mechanical properties and weldability of Fe-Cr-Mo with Mo content higher than used in commercial alloys. In this study, pseudo-binary diagrams for different compositions of Fe-Cr-Mo alloys were made to identify the phases and possible heat treatments. Solubilized samples of different alloy compositions were characterized by optical microscopy. Welds were carried out with and without the addition of material using TIG (Tungsten Inert Gas) and MIG (Metal Inert Gas) processes and tge Heat-Affected Zone grain sizes of alloys and microstructural characterization of welded regions in various parts of the sample were analyzed.

After analyzing different points of the sample of HAZ, the results showed that the alloys Fe-Cr-Mo showed a ferritic microstructure in the solubilized condition, with large grains and mechanical properties superior to commercial alloys.

The analysis of the HAZ is performed entirely in the optical microscope, from its origin to the end, for a thorough analysis. This analysis is only considered a local analysis. When it is necessary to perform a full analysis of the sample, the expert must capture several images sequentially in the optical microscope and join the images together to hold a macro analysis. This analysis is useful, however, the high cost of time (labor hours) often obstructs such an analysis by experts. Therefore, for the tests in this article, we use the images of 16 different samples. For each sample, several images were collected of the different regions of the sample, as shown in Table 1.

**Table 1:** Data from the 16 samples used in this study.

Sample Number	Width Image	Height Image	Number of Images
1	398	2600	5
2	408	3400	7
3	408	220	4
4	400	3400	7
5	408	220	6
6	396	3400	7
7	401	3000	6
8	402	2200	4
9	403	2600	6
10	406	2200	4
11	400	3400	7
12	398	2600	6
13	403	300	6
14	403	1800	3
15	398	2600	5
16	404	3000	6

**Figure 2:**
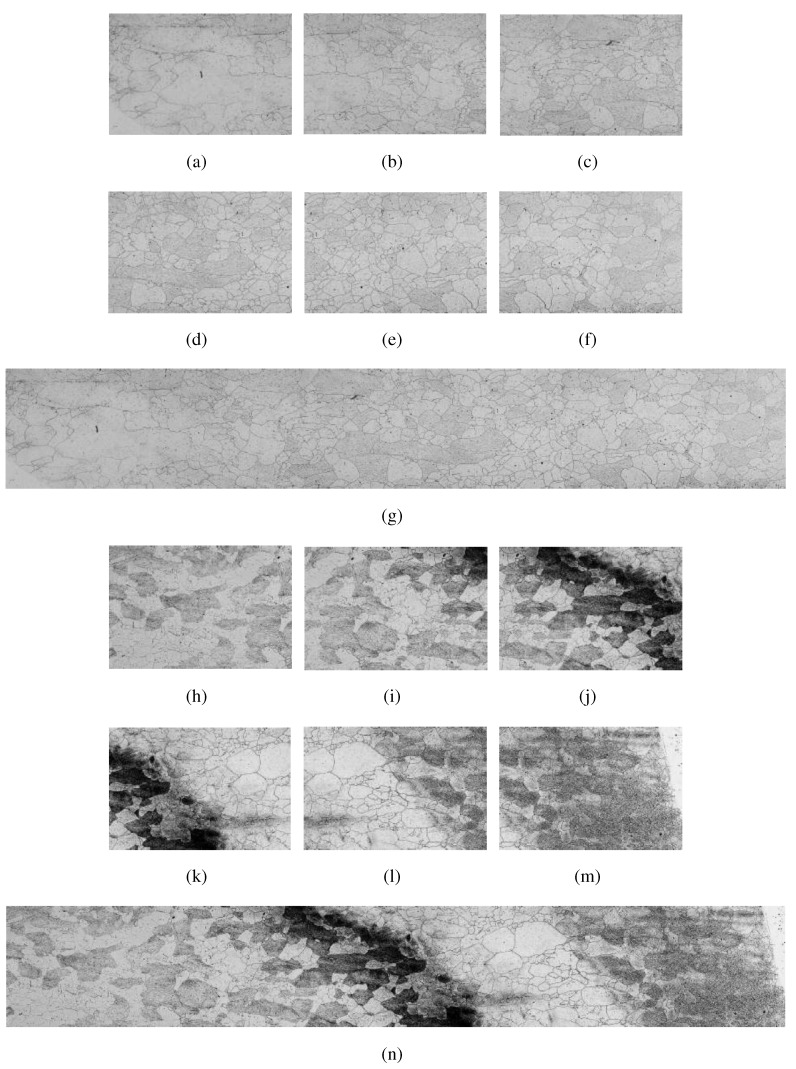
Example manual mosaic construction by a specialist. (**a**)–(**f**) images of sample 9 from database, generating the mosaic (**g**). (**h**)–(**m**) images of sample 12 from database, generating the mosaic (**n**).

Figure 2 shows an example of manual mosaic construction by a specialist, taken from samples 9 and 12 of the database.

To allow a better analysis of all the sample region, the use of the mosaic technique is submitted. This method allows the construction of a larger image from smaller images with corresponding characteristics obtained through the use of the microscope,and with these characteristics the necessary transformations are performed [13]. Several methods and computer systems were developed for the construction of the mosaic from microscopic images. Among them are methods used for the diagnostic of the medical applications with the capability of constructing the mosaic from microscopic images, systems for stitching several images obtained from microscope [14,15]. This paper proposes the development of the specific system to aid both the researcher and laboratory's analyst, because many laboratories do not have any systems to aid in their analysis. Therefore, this proposed system can improve the capability of analysis and obtain results quickly by the specialists.

**Figure 3:**
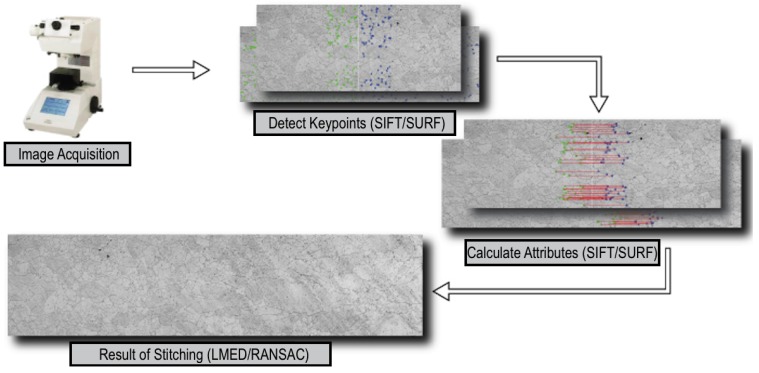
Steps of the process of analysis proposed through of the mosaic construction using SIFT and SURF method.

In the Figure 3 is show the steps of the process of analysis proposed through of the mosaic construction using the SIFT and SURF method, transformed with the images obtained from an optical microscope.

### 2.1. Mosaic Construction Using Transformed Scale Invariant Feature Transform

The transformed Scale Invariant Feature Transform is an important computational tool used to find corresponding points between images [16]. In geo-processing, some techniques of Digital Image Processing are already used, several of them use the transform, SIFT, as [17], whose primary focus of work, optimizes the alignment error of the images.

The transformed SIFT is an important computational tool used to find corresponding points between images [16]. In geo-processing, some techniques of digital image processing are already used, several of them use the transform SIFT as [17], whose primary focus of his work, optimize the alignment error of the images.

**Figure 4:**
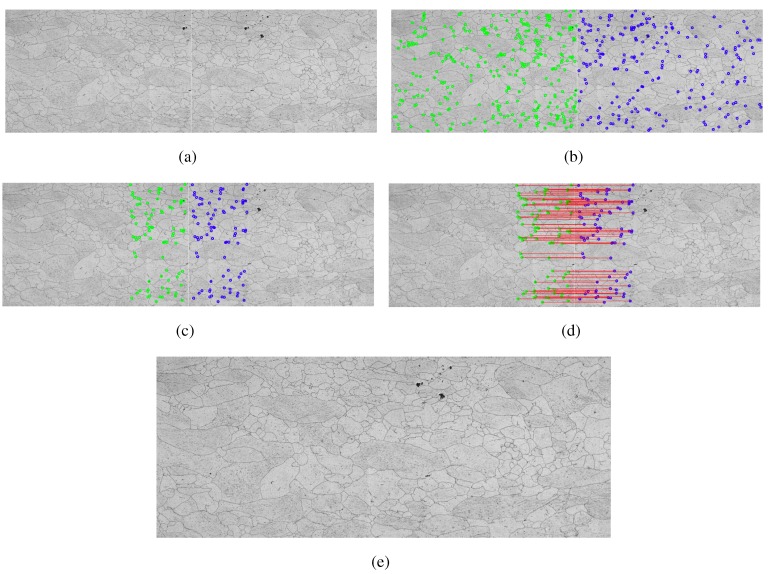
Example of the step by step construction using Transformed SIFT, (**a**) two adjacent frames with common points; (**b**) key points located by the Transformed SIFT; (**c**) choice of possible candidates for common points; (**d**) calculation of the nearest neighbor, and end result, grouped images; (**e**) mosaic built.

In this method, the construction tool of the mosaics is based on the use of three complementary techniques as follows [18]: Scale Invariant Feature Transform, responsible for extracting image characteristics, and Random Sample Consensus (RANSAC), used in the filtering of false points between corresponding images. Figure 4 shows the application of this method in metallographic images.

The SIFT extracts specific characteristics of images or of certain regions and stores them in local descriptors [16]. The characteristics of these descriptors do not suffer variation compared to scaling and rotation and are used in the comparison of the images. These descriptors form vector-specific information points and has distinct features allowing the analyst to find points in large databases with high precision.

After locating the descriptors, the analyst will need to find common points in the two images, this correlation is performed by calculating the nearest neighbor (KNN), which uses the minimum Euclidean distance between the possible points in common.

This system also has a tool to filter out potential false correlations, which are indicated as corresponding points, but that are not equal. This is the RANSAC function that calculates the number of correct correlations in the data matrix, minimizing errors [18].

After the correct determination of the common points between the images, you can perform the necessary transformations and connect the images to form a single larger image.

### 2.2. Mosaic Construction Using Transformed Speeded Up Robust Features

The Speeded Up Robust Features descriptor is a method for computing distinctive invariance local features quickly [19]. It is an efficient implementation of the SIFT descriptor and is more faster than a SIFT descriptor for variations in deformations such as image rotation, image blur, light changes, scale changes, JPEG-compression and viewpoint changes [20].

SURF descriptor using a determinant of the Hessian matrix, detects feature points [21] and consists of two principal parts [20], Detector and Descriptor. Detector reduces significantly computation time using an integral image, Hessian matrix-based interest points, scale-space representation and interest point localization. For a Descriptor, every interest point sought by the detector has to carry its own indicator to assign invariability to the interest points. When deformations occur in an image, the interest point descriptors can be employed to look for correlations between the original image and the transformed image.

**Figure 5:**
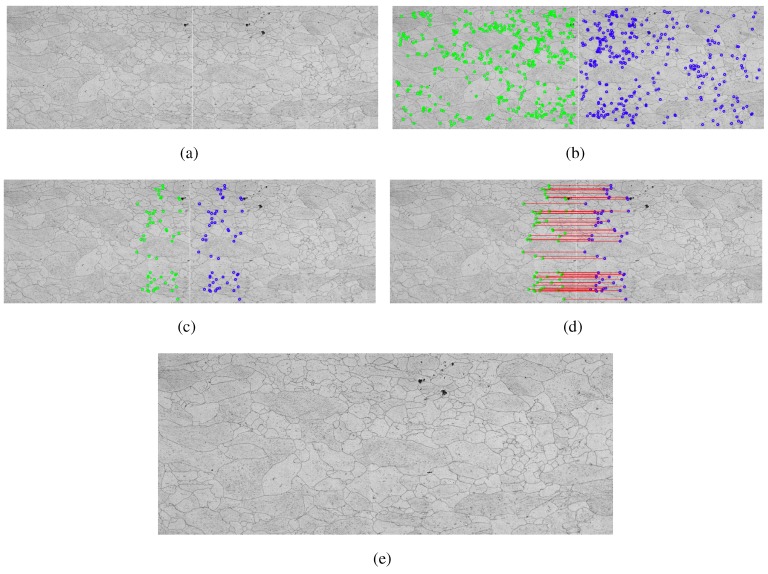
Example of the step by step construction using Transformed SURF, (**a**) two adjacent frames with common points; (**b**) key points located by the Transformed SURF; (**c**) choice of possible candidates for common points; (**d**) calculation of the nearest neighbor; and (**e**) the end result of grouped images.

Figure 5 shows an example of the application of the SURF method in metallographic images.

The Hessian matrix H(x,σ) which is defined as follows:

(1) $$H\left(x,\sigma \right)=\left[\begin{array}{cc} L{}_{xx}\left(x,\sigma \right) & L{}_{xy}\left(x,\sigma \right)\\ L{}_{xy}\left(x,\sigma \right) & L{}_{yy}\left(x,\sigma \right) \end{array}\right]$$

where L is the convolution of the Gaussian second order derivation of image at point H(x,y) in scale and similarly for L(x,y) and L(y,y).

The Scale-Space is constructed using box filters, instead of Gaussian filters, which are used in SIFT. These box filters can be evaluated very quickly using integral images, independent of size. To localize interest points in the image and over scales, a non-maximal suppression in a 3×3×3 neighborhood is applied. Then, the nearby data is interpolated to find the location in both space and scale to sub-pixel accuracy. Extraction of the descriptor consists of fixing a reproducible orientation based on information from a circular region around the interest point. Then a square region is built to align the selected orientation, and extract the SURF descriptor from it [22].

### 2.3. Variations in the Construction of Mosaics with SIFT and SURF

The SIFT and SURF methods find the key points and change the perspectives of the images to join them. To change the perspective of the image, a homography matrix must be found which will be used to generate the perspective of the new image. The RANSAC and Least-Median (LMED) methods can be used to find the homography matrix.

In RANSAC, subsets of the input data are randomly selected and model parameters fitting the sample are computed repeatedly. In the next step, the quality of the parameters is evaluated from the input data (number of inliers). The process ends when the probability of finding a better model becomes lower than a user-controlled probability [23].

LMED method consists of choosing a number of pixels randomly from the local window surrounding the pixel where a motion estimate needs to be carried out. A temporary solution is calculated from the number of linear independent equations for the chosen pixels. The residual error at each pixel in the local window is calculated using the temporary solution for the parameters. One then finds the median of the square of the error values. This process is repeated several times and then the set of parameters that corresponds to the minimum of the medians is chosen as the final solution [24].

The homography matrix is used to distort images, generating a new perspective to join them. This process is necessary to approximate the pixels that were distorted. The approximation is accomplished using interpolation methods. There are various image interpolation techniques, but in this paper, two were tested: Linear Interpolation and Cubic Interpolation.

For separated bi-linear interpolation, the values of both direct neighbors are weighted by their distance to the opposite point of interpolation. The main disadvantages of Linear Interpolation are both the attenuation of high-frequency components and the analyzing of the data beyond the cut-off point into the low frequencies. Cubic polynomials are used frequently because of their ability to fit C2-continuous. Cubic polynomials also can be used to approximate the sync function. In the case of Cubic Interpolation, with two points the resulting curves are similar to those obtained by linear interpolation, but the pieces fit C1-continuously in the spatial domain [25].

**Figure 6:**
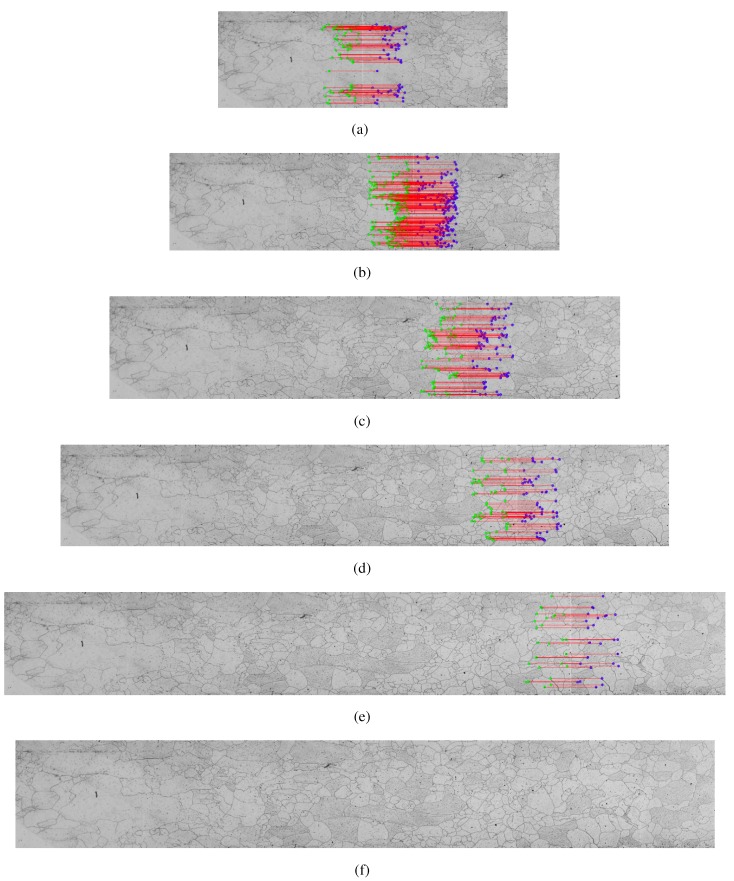
Mosaic construction example from the SIFT method using RANSAC Homography method to find the matrix and the Linear Interpolation method for constructing mosaic images using the five images collected by the microscope—sample 12, (**a**) first step, mosaic of the two initial images; (**b**)–(**e**) mosaic previous result with an adjacent image; (**f**) final result.

A mosaic construction example from the SIFT method is shown in Figure 6. In the example, we use the RANSAC method to find homography matrix. The linear interpolation method is used to construct the mosaic with five images of the sample 12.

**Figure 7:**
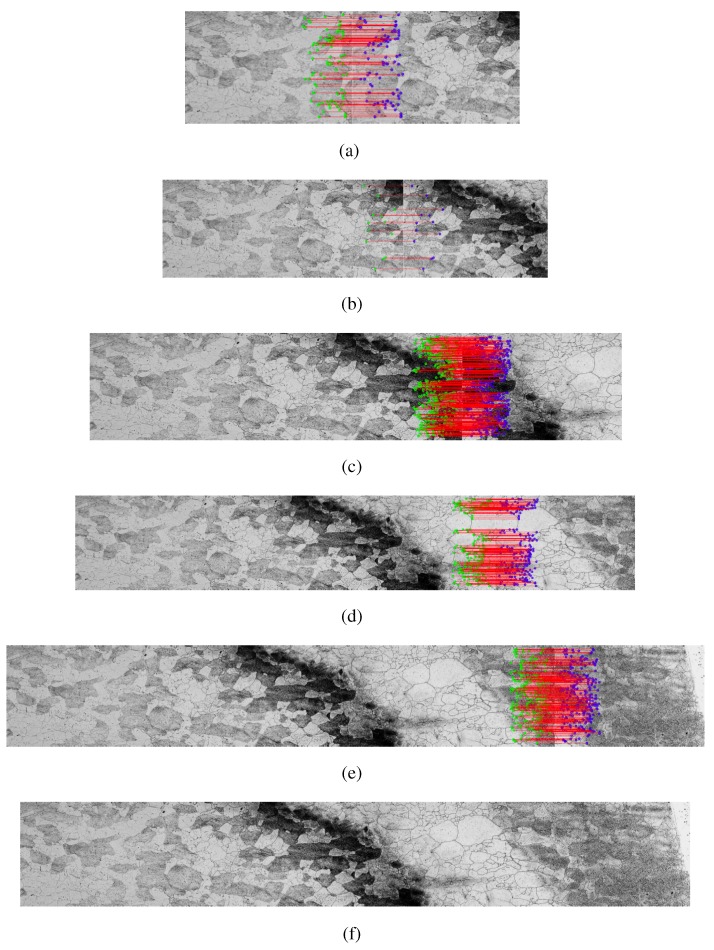
Mosaic construction example from the SURF method using LMED Homography method to find the matrix and the Linear Interpolation method for constructing mosaic images using the five images collected by the microscope—sample 9, (**a**) first step, mosaic of the two initial images; (**b**)–(**e**) mosaic previous result with an adjacent image; (**f**) final result.

The Figure 7 shows the results of applying the transformed SURF in building the mosaic. The mosaic was built using the Linear Interpolation method in five images of the sample 9. The homography matrix was found using the LMED method.

The Figure 6a and Figure 7a show the first two images of each method with its key points in common found by SIFT and SURF transformed, respectively. The result of the merger of the two images is shown on the left of the Figure 6b and Figure 7b. The results are shown in the following images, adding each new image to a mosaic that result in the mosaics of Figure 6f and Figure 7f.

### 2.4. Variables in Mosaic Quality Measurements

The metrics to evaluate mosaics are used in image registration when is necessary to measure the similarity between images, because provides a value that should be use for measurement of similarity between the mosaic image and the input images/frames [26,27,28,29]. According to [30] the metrics evaluate the quality images and to measure the degradation in digital images in order to improve the quality of the resultant image. To assess the quality of the mosaic construction, the following metrics were used: Mean Squared Error (MSE), Peak Signal-to-Noise Ratio (PSNR) and Mean Structural Similarity Index (MSSIM).

The MSE represents the power of the difference between original and distorted images. PSNR is a logarithmic representation of the MSE, and it is usually expressed in terms of the logarithmic decibel where 255 is the maximum possible amplitude for an 8-bit image. High values of the PSNR, define an improved quality of reproduction [31].

The Structural SIMilarity (SSIM) metric is a perceptual metric based on the content features of extraction and abstraction. The structure of the objects in the scene can be represented by its attributes, which are independent of both contrast and average luminance. Therefore, the changes in the structural information from the reference and distorted images, can be perceived as a measure of the image distortion. The MSSIM value is the mean value of SSIM map over the whole image [31].

These metrics will be used for comparative measurements between the mosaics obtained by the proposed methods.

## 3. Results and Discussion

In all, database images were applied using SIFT and SURF techniques with variations, LMED and RANSAC, to determine the homography matrix, and Linear and Cubic Interpolation to construct the mosaic.

**Figure 8:**
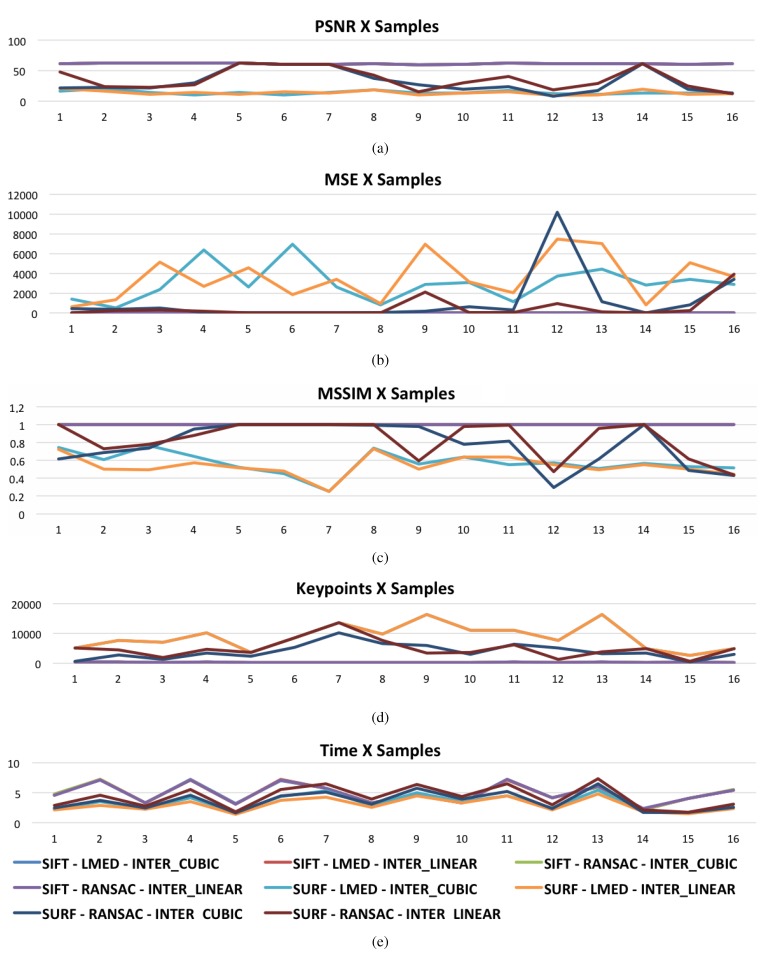
Measure of SIFT and SURF variations: (**a**) PSNR; (**b**) MSE; (**c**) MMSIM; (**d**) Number of keypoints and (**e**) time in seconds.

Figure 8 shows the graphs with the measures of adopted metrics for variations of SIFT and SURF methods. The Figure 8a shows the value of the metric PSNR, Figure 8b of the metric MSE and Figure 8c of the MSSIM metric for the 16 samples. It is observed that for all variations of the SIFT method, the metrics adopted as quality measures of the mosaics remain constant. Using the SURF method the metrics already have many changes. Figure 8d shows the amount of key points found in each sample for variations of the methods used. Figure 8e shows a mosaic construction time chart for all variations utilized. From the graphic, it is quite evident that variations in the SURF method are much faster than the SIFT method. In some instances, the SIFT method is twice as slow as the SURF method.

By doing a comparison of the graphs in Figure 8e,d, it is noticed that with a greater number of keypoints in common, the faster the building of the mosaic becomes. In all variations of the SIFT method there is a constant quantity of keypoints found. A constancy that is not observed in the variations of the SURF method.

Table Table 2 shows the mean and standard deviation for the SIFT and SURF variations of PSNR, MSE and MMSIM measures, and also the mosaic construction time and found keypoints. The image dimensions are 600×400 pixels, and it was used in the experiments a PC Intel Core i5 1,4 GHz, with 4 GB of RAM, in Operational System MAC OS X 10.9.5.

As shown in Table 2, even with the large variation in the values of the metrics results, the SURF method is faster than the SIFT method. Although it has a higher speed in building the mosaic, its speed does not compensate for errors generated by the method.

**Table 2:** Mean and standard deviation for variations of SIFT and SURF of PSNR measures, MSE and MMSIM, in wich *R* means RANSAC and *M* means LMED to find Homography Matrix, and *C* and *L* means Cubic and Linear Interpolation.

SIFT	PSNR	MSE	MSSIM	Seconds	Keypoints
M - C	61.38 ± 0.851	0.048 ± 0.009	0.999 ± 3.5E-05	4.92 ± 1.67	326.9 ± 70.01
M - L	61.38 ± 0.851	0.048 ± 0.009	0.999 ± 3.5E-05	4.91 ± 1.69	326.9 ± 70.01
R - C	61.38 ± 0.851	0.048 ± 0.009	0.999 ± 3.5E-05	4.93 ± 1.68	326.9 ± 70.01
R - L	61.38 ± 0.851	0.048 ± 0.009	0.999 ± 3.5E-05	4.91 ± 1.65	326.9 ± 70.01
SURF	PSNR	MSE	MSSIM	Seconds	Keypoints
M - C	14.19 ± 3.040	3007.20 ± 1786.17	0.573 ± 0.1251	3.34 ± 1.30	8792.0 ± 4204.2
M - L	13.73 ± 3.477	3548.32 ± 2301.38	0.536 ± 0.1142	2.98 ± 1.13	8794.9 ± 4207.2
R - C	31.68 ± 18.844	1118.01 ± 2561.57	0.773 ± 0.2329	3.58 ± 1.55	3993 ± 2536.5
R - L	36.19 ± 17.682	504.07 ± 17.68	0.840 ± 0.427	4.25 ± 1.85	4926 ± 3120.7

## 4. Conclusions

This paper proposes the creation of mosaics for performing metallographic analysis through Digital Image Processing. For this the SIFT and SURF methods are used. For the methods adopted, variations were utilized to find homography matrix (LMED and RANSAC methods) and for joining the images for generation of the mosaics (both Linear and Cubic Interpolation). The methods, with variations, were applied in 16 samples of metallographic images.

Based on experimental results, the variation of the transformed SIFT did not show variations in quality metric measurement of the mosaics. On average, the values of metric and standard deviation, for this method, are: PSNR = 61.38 ± 0.852, MSE = 0.048 ± 0.009 and MSSIM = 0.999 ± 3.5 × 10 ${}^{-5}$. The average time of creation of the mosaics to the variations of SIFT method, is 4.91 s. The average of keypoints found is 326.9.

Already, the variations of the transformed SURF demonstrate changes in the measured quality metrics of the mosaics and also a larger number of keypoints. The largest amount of keypoints caused by the variations of SURF method show faster results than the SIFT method, while this variation in metrics of the SURF method show that they have a greater distortion in the mosaics.

At the end of the testing, it was concluded that although it is a slower process, the SIFT method is more stable and has a better performance than the SURF method and it can be applied to real applications.

In future work, we suggest application of changes for the methods proposed in more samples to conduct a performance evaluation of methods with a greater range of data. The use of other techniques to find homographic matrix and perform the union of the images to create of the mosaics is also proposed. We intend to apply image enhancement techniques such as a pre-processing step to evaluate performance, as proposed by [32].

The methodology presented opens a new frontier in the analysis of macroscopic samples using optical microscopy images. Therefore, this approach can and should be used in commercial systems.
